# Combined Regional Analgesia for Complex Unilateral Clavicular and Thoracic Trauma Undergoing Clavicle Fixation: A Case Report

**DOI:** 10.7759/cureus.112487

**Published:** 2026-07-11

**Authors:** Rahul Kalshan, Trisha Nidhi, Varsha Jakhar, Mamta Agarwal, Dhirja Sharma

**Affiliations:** 1 Anaesthesia, Safdarjung Hospital, New Delhi, IND; 2 Anaesthesiology, Yatharth Super Speciality Hospital, Model Town, New Delhi, IND; 3 Anaesthesiology, Holy Family Hospital, New Delhi, IND

**Keywords:** anesthesia and analgesia, interests in difficult airway and regional anesthesia, post-anesthesia care unit (pacu), pre-anesthesia check-up, surgical anesthesia

## Abstract

Patients with simultaneous clavicular and ipsilateral thoracic trauma, including multiple rib fractures and traumatic pneumothorax, present a complex perioperative analgesic challenge. Effective pain control must be balanced against the preservation of respiratory function while minimizing opioid-related adverse effects. Individual regional anesthesia techniques for clavicle surgery and rib fracture analgesia are well established; however, selecting an appropriate multimodal analgesic strategy for patients with combined injuries requires careful clinical judgment.

We report the case of a 51-year-old man who sustained a displaced right midshaft clavicle fracture, multiple right-sided rib fractures, a comminuted scapular fracture, and a small traumatic pneumothorax following blunt chest trauma. The patient underwent open reduction and internal fixation (ORIF) of the clavicle under general anesthesia. After securing the airway with endotracheal intubation, an ultrasound-guided right erector spinae plane (ESP) block using 20 mL of 0.25% bupivacaine was performed to provide analgesia for the rib fractures and chest wall injury, followed by an ultrasound-guided right interscalene block using 20 mL of 0.25% bupivacaine for clavicular surgical analgesia as part of a multimodal analgesic strategy.

The intraoperative course remained hemodynamically stable without significant fluctuations in heart rate or blood pressure. Postoperatively, the patient experienced excellent analgesia without immediate rescue opioid requirements, maintained satisfactory respiratory function, and was able to perform deep breathing, effective coughing, and early mobilization.

This case highlights the importance of individualized perioperative anesthetic planning in patients with complex unilateral clavicular and thoracic trauma. Although the regional anesthesia techniques employed are well established, their combined application should be guided by the injury pattern, respiratory considerations, and the need to provide effective targeted analgesia for distinct anatomical regions.

## Introduction

Clavicle fractures account for approximately 2-5% of all fractures and are frequently associated with high-energy trauma. Concurrent thoracic injuries, particularly multiple rib fractures, significantly increase perioperative morbidity because of severe pain, impaired respiratory mechanics, reduced cough effectiveness, and an increased risk of pulmonary complications [1,2].

Adequate analgesia is a cornerstone of management in patients with rib fractures. Inadequate pain control results in splinting, hypoventilation, ineffective cough, secretion retention, atelectasis, and pneumonia, all of which contribute to increased respiratory morbidity [2,3]. Although systemic opioids remain widely used, opioid-induced sedation and respiratory depression may further compromise pulmonary function in patients with chest trauma, highlighting the importance of opioid-sparing regional analgesic techniques [3].

The erector spinae plane (ESP) block, first described by Forero et al. in 2016, has emerged as an effective regional analgesic technique for thoracic and chest wall pain, including traumatic rib fractures [4]. By providing multilevel thoracic analgesia through the spread of local anesthetic along the erector spinae fascial plane, the ESP block offers effective pain relief while avoiding some of the limitations associated with thoracic epidural analgesia [5].

Analgesia for clavicle surgery presents a distinct challenge because sensory innervation is derived from both the superficial cervical plexus and the brachial plexus. Ultrasound-guided interscalene block is a well-established regional anesthetic technique that provides reliable perioperative analgesia for clavicular and shoulder procedures [6,7].

Patients with simultaneous clavicular and ipsilateral thoracic trauma, including multiple rib fractures, scapular fractures, and traumatic pneumothorax, present a unique perioperative analgesic challenge. Effective pain management must address injuries arising from different anatomical regions while preserving respiratory function in a patient already at risk of pulmonary complications. The educational value in such cases lies not in the individual regional anesthesia techniques themselves, but in the clinical decision-making required to tailor analgesia to a complex injury pattern. We report the perioperative anesthetic management of a patient with unilateral clavicular and thoracic trauma undergoing open reduction and internal fixation (ORIF) of the clavicle, in whom a combined ultrasound-guided ESP and interscalene block strategy was incorporated into a multimodal anesthetic approach to provide targeted analgesia while minimizing opioid requirements.

## Case presentation

A 51-year-old man presented to Holy Family Hospital following a fall from the stairs while under the influence of alcohol. He sustained blunt trauma to the right side of the chest and shoulder. The patient was a chronic alcohol consumer with no significant medical comorbidities or history of previous cardiopulmonary disease.

On physical examination, there was marked tenderness over the right clavicle and right chest wall. Respiratory examination revealed decreased air entry over the right hemithorax. Airway assessment was unremarkable, with adequate mouth opening, normal neck movements, and no predictors of difficult airway. Baseline oxygen saturation ranged between 96% and 99% on room air.

Preoperative laboratory investigations are summarized in Table 1. Electrocardiography demonstrated a normal sinus rhythm with right bundle branch block. Transthoracic echocardiography showed preserved left ventricular systolic function with an ejection fraction of approximately 55% and no regional wall motion abnormalities.

**Table 1:** Preoperative laboratory investigations

Investigation	Result	Reference range
Hemoglobin	14.2 g/dL	13-17 g/dL
Hematocrit	42%	40-50%
Total leukocyte count	10,000/mm³	4,000-11,000/mm³
Platelet count	212×10³/mm³	150-450×10³/mm³
Random blood sugar	99 mg/dL	70-140 mg/dL
Blood urea nitrogen	20 mg/dL	7-20 mg/dL
Serum creatinine	0.76 mg/dL	0.6-1.2 mg/dL
Sodium	141 mmol/L	135-145 mmol/L
Potassium	3.87 mmol/L	3.5-5.0 mmol/L
Chloride	109.2 mmol/L	98-107 mmol/L
Prothrombin time	10.2 sec	10-13 sec
International normalized ratio	0.91	0.8-1.2
Activated partial thromboplastin time	27 sec	25-35 sec

**Figure 1:**
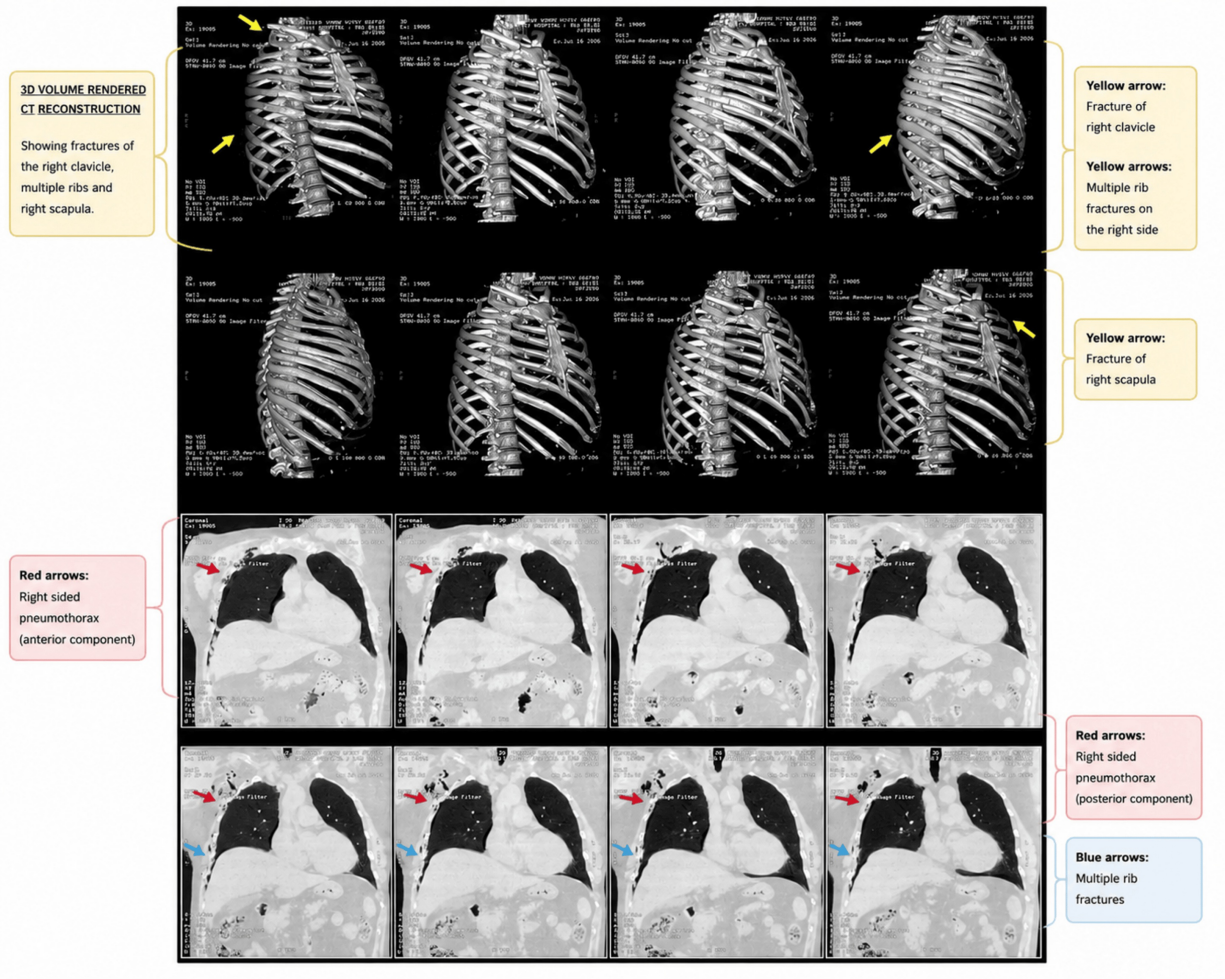
Preoperative CT of the thorax demonstrating the traumatic injuries (a-h) Three-dimensional volume-rendered CT reconstructions demonstrating a displaced fracture of the middle third of the right clavicle (yellow arrow), multiple right-sided rib fractures (yellow arrows), and a comminuted fracture of the right scapular body (yellow arrow). (i-p) Coronal lung-window CT images demonstrating a mild right-sided traumatic pneumothorax (red arrows) with associated subcutaneous emphysema and multiple right-sided rib fractures (blue arrows). CT: computed tomography

Computed tomography (CT) of the thorax demonstrated a displaced fracture of the middle third of the right clavicle, multiple right-sided rib fractures, a comminuted fracture of the right scapular body, and a mild right-sided traumatic pneumothorax with ipsilateral subcutaneous emphysema, and three-dimensional volume-rendered CT reconstruction further delineated the osseous injuries (Figure 1). The imaging findings demonstrated the complexity of the unilateral injury pattern and were considered during perioperative planning to optimize analgesia while minimizing respiratory compromise.

After multidisciplinary discussion involving the orthopedic, anesthesia, and critical care teams, the patient was scheduled for ORIF of the right clavicle.

Standard American Society of Anesthesiologists (ASA) monitoring, including electrocardiography, pulse oximetry, non-invasive blood pressure monitoring, and capnography, was instituted. General anesthesia was induced with intravenous fentanyl, propofol, and rocuronium, following which the trachea was intubated uneventfully.

**Figure 2:**
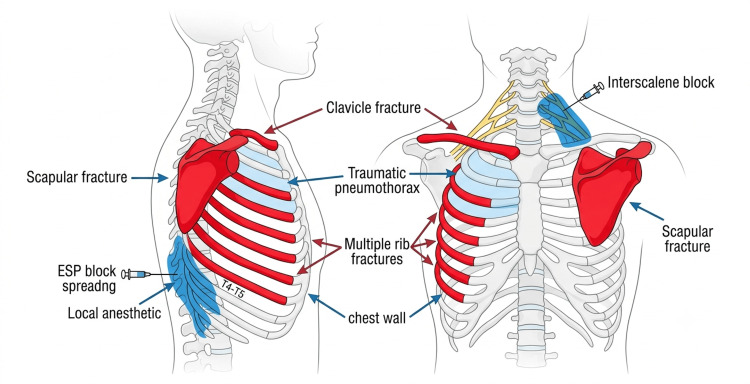
Schematic illustration demonstrating the anatomical relationship between the right clavicular fracture, multiple ipsilateral rib fractures, scapular fracture, and right traumatic pneumothorax together with the anatomical targets of the regional anesthesia techniques. The ultrasound-guided ESP block was used to provide analgesia for the rib fractures and chest wall injury, whereas the ultrasound-guided interscalene brachial plexus block was used to provide perioperative analgesia for clavicle fixation ESP: erector spinae plane This image was created using Canva (Canva Pty Ltd., Sydney, New South Wales, Australia).

After securing the airway, an ultrasound-guided right ESP block was performed using 20 mL of 0.25% bupivacaine to provide targeted analgesia for the multiple rib fractures and chest wall injury. This was followed by an ultrasound-guided right interscalene brachial plexus block using 20 mL of 0.25% bupivacaine to provide analgesia for the clavicular fracture. The airway was intentionally secured before performing the regional blocks because positive-pressure ventilation was anticipated during surgery and the presence of a traumatic pneumothorax warranted controlled intraoperative respiratory management. A combined regional analgesic strategy was selected to address pain arising from distinct anatomical regions while minimizing perioperative opioid requirements and facilitating postoperative respiratory function. The anatomical relationship between the injury pattern and the regional anesthesia techniques used is illustrated schematically (Figure 2).

Anesthesia was maintained with sevoflurane in an oxygen-air mixture. Intravenous paracetamol, tramadol, dexamethasone, and ondansetron were administered as part of a multimodal analgesic regimen.

The surgical procedure was completed uneventfully. Intraoperative hemodynamic parameters remained stable without significant fluctuations in heart rate or blood pressure, and no additional opioid supplementation was required beyond routine anesthetic management.

At the completion of surgery, residual neuromuscular blockade was reversed, and the trachea was extubated successfully. The patient reported excellent postoperative analgesia involving both the clavicular operative site and the injured chest wall. Adequate pain control facilitated deep breathing, effective coughing, and early mobilization. No immediate postoperative respiratory complications were observed, and the postoperative recovery was uneventful.

## Discussion

This case highlights the anesthetic decision-making required in patients with complex unilateral clavicular and thoracic trauma, where effective analgesia must be balanced against the preservation of respiratory function.

Rib fracture analgesia and respiratory implications

Multiple rib fractures are associated with substantial morbidity due to severe pain and impaired respiratory mechanics [1,2]. Inadequate analgesia may result in hypoventilation, secretion retention, atelectasis, and pneumonia [2,3]. Consequently, effective pain control remains a key component of management.

Thoracic epidural analgesia has traditionally been considered the gold standard for severe rib fracture pain; however, technical difficulty, hypotension, and contraindications limit its application in many trauma patients [8]. Fascial plane blocks such as the ESP block have therefore gained increasing popularity owing to their simplicity and favorable safety profile [5].

The ESP block provides analgesia through the cranio-caudal spread of local anesthetic beneath the erector spinae muscle, producing multilevel thoracic analgesia [4,5]. Several reports have demonstrated its effectiveness in patients with traumatic rib fractures and thoracic pain syndromes [4,9].

Clavicle fracture analgesia

The clavicle receives sensory innervation from both the cervical and brachial plexuses, making regional anesthesia for clavicle surgery particularly challenging [6]. Interscalene block remains one of the most commonly utilized techniques because it provides reliable analgesia to the shoulder girdle and clavicular region [6,7].

In our patient, the interscalene block was specifically selected to provide perioperative analgesia for clavicle fixation, while the ESP block was directed toward analgesia of the fractured ribs and chest wall. This dual-block approach enabled targeted coverage of both injury sites.

Regional anesthesia in the presence of traumatic pneumothorax

The coexistence of a traumatic pneumothorax further complicated anesthetic planning because effective analgesia had to be balanced against the preservation of respiratory function. Adequate pain control was particularly important to facilitate deep breathing, effective coughing, and postoperative pulmonary hygiene. The airway was intentionally secured before performance of the regional blocks because positive-pressure ventilation was anticipated during surgery, while the presence of a traumatic pneumothorax warranted careful intraoperative respiratory monitoring. This multimodal analgesic strategy permitted satisfactory perioperative respiratory function without clinical evidence of respiratory deterioration.

No clinically significant respiratory deterioration occurred during the perioperative period. Furthermore, excellent postoperative analgesia enabled effective respiratory effort and patient comfort.

Significance of the combined approach

Reports describing the combined use of ESP and interscalene blocks in patients with clavicle fracture, multiple ipsilateral rib fractures, scapular fracture, and traumatic pneumothorax remain limited. This case demonstrates how targeted regional anesthesia techniques can be integrated into a multimodal analgesic strategy to provide comprehensive perioperative pain control while minimizing opioid exposure.

## Conclusions

Patients with concomitant unilateral clavicular and thoracic trauma present a complex perioperative analgesic challenge because effective pain control must be balanced against the preservation of respiratory function. This case highlights the importance of individualized anesthetic planning and demonstrates that a combined ultrasound-guided ESP and interscalene block strategy can provide effective targeted analgesia as part of a multimodal anesthetic approach in carefully selected patients. Although both regional anesthesia techniques are well established, their combined application may be particularly useful in patients with ipsilateral clavicular and thoracic injuries requiring analgesia for distinct anatomical regions while minimizing perioperative opioid requirements. Further studies are needed to evaluate the safety and effectiveness of this approach in larger patient populations.
